# Therapeutic strategies for adrenocortical carcinoma: integrating genomic insights, molecular targeting, and immunotherapy

**DOI:** 10.3389/fimmu.2025.1545012

**Published:** 2025-03-12

**Authors:** Jing Sun, Jiaxuan Huai, Wenhui Zhang, Tianyu Zhao, Run Shi, Xuanbin Wang, Minglun Li, Xuehua Jiao, Xiqiao Zhou

**Affiliations:** ^1^ Department of Endocrinology, Jiangsu Province Hospital of Chinese Medicine, Affiliated Hospital of Nanjing University of Chinese Medicine, Nanjing, China; ^2^ The First School of Clinical Medicine, Nanjing University of Chinese Medicine, Nanjing, China; ^3^ Institute and Clinic for Occupational, Social and Environmental Medicine, Ludwig Maximilians University (LMU) University Hospital Munich, Munich, Germany; ^4^ Department of Oncology, The First Affiliated Hospital of Nanjing Medical University, Nanjing, China; ^5^ Laboratory of Chinese Herbal Pharmacology, Department of Pharmacology, Hubei Key Laboratory of Wudang Local Chinese Medicine Research, Renmin Hospital, Hubei University of Medicine, Shiyan, Hubei, China; ^6^ Department of Radiation Oncology, Lueneburg Hospital, Lueneburg, Germany; ^7^ Department of Endocrinology, Suzhou Ninth People’s Hospital, Suzhou Ninth Hospital Affiliated to Soochow University, Suzhou, China

**Keywords:** adrenocortical carcinoma, molecular pathogenesis, targeted therapies, immunotherapy, genomic insights

## Abstract

Adrenocortical carcinoma (ACC) is an uncommon and highly aggressive cancer originating in the adrenal cortex, characterized by a high likelihood of recurrence and unfavorable survival rates, particularly in the advanced disease stages. This review discusses the complex molecular pathogenesis of ACC, focusing on critical pathways implicated in the tumorigenesis and providing potential targets for therapy: the Wnt/β-catenin signaling pathway, the IGF2/IGF1R axis, and the apoptosis pathway regulated by p53. Current treatment strategies include surgical resection and mitotane, the sole adrenolytic agent approved by the FDA; however, its effects in advanced disease are suboptimal. Cytotoxic chemotherapy combined with mitotane may be applied, but survival benefits are limited so far. In the following review, we outline emerging targeted therapies, such as mTOR inhibitors and tyrosine kinase inhibitors (TKIs), which show favorable preclinical and clinical data, especially in treatment-resistant ACC. We also emphasize the possible role of immune checkpoint inhibitors (ICIs) in the management of ACC, although their effectiveness is still under study. Upcoming trends in treatment involve forms of personalized medicine, where molecular profiling is integrated to identify actionable biomarkers for administered therapies. This review will attempt to provide a comprehensive framework on how recent breakthroughs in the genomics of ACC, coupled with advances in targeted therapies and immunotherapy, can improve management.

## 1 Introduction

Adrenocortical carcinoma (ACC) is a rare and highly invasive cancer emerging from the adrenal cortex, with an estimated incidence rate of about 0.7-2 cases per million individuals globally each year (1–5). The age profile of ACC follows a bimodal pattern with significant peaks in early childhood, around age 5, and reoccurring in the age range of 40s to 50s (1, 6, 7). The survival rate over a 5-year period for ACC varies significantly by stage, ranging from approximately 60-80% for tumors localized, 35-50% for locally advanced cases, and less than 30% for those with distant metastasis (5, 8). For localized tumors treated with surgery and adjuvant therapy, survival rates ranged from 75% to 82% (7, 9). Recurrence after curative surgical treatment remains a significant challenge, with recent studies reporting a recurrence rate of 70% among patients with stage I-III and a median disease-free survival (DFS) duration lasting 11 months following surgery (10). Pediatric patients generally show better outcomes, especially those aged 4 years or younger, with survival rates ranging from 54.2% to 91% (9, 11).

ACC prognosis is influenced by various clinical factors, including patient age, tumor stage, hormone secretion, tumor grade, Ki-67 index, somatic gene mutations, methylation profiles, microRNA and gene expression patterns, and tumor margin status (9). Complete tumor resection through surgery remains the sole curative option for ACC. However, recurrence rates vary significantly depending on the tumor grade, with lower-grade tumors having a recurrence rate of approximately 20-30%, while higher-grade tumors can have recurrence rates exceeding 70%. Due to these high rates of recurrence, the use of adjuvant treatment is critical to improve long-term outcomes and reduce the likelihood of cancer recurrence. Mitotane is the single drug approved by the Food and Drug Administration (FDA), utilized both as adjuvant therapy and in advanced disease stages (12, 13). However, the treatment options for ACC are often limited and insufficient, especially in advanced stages, due to its heterogeneous nature, which leads to varied responses to conventional therapies. First-line treatment (EDP-M), which involves mitotane along with etoposide, doxorubicin, and cisplatin, shows low response rates, with approximately 1.3% of patients attaining a complete response (CR), while 19.2% show a partial response (PR) (5, 14). Given the unsatisfactory outcomes of current therapies, an urgent need exists for novel treatment options to enhance survival rates, reduce recurrence risk, and address drug resistance.

Recent advances in whole-genome molecular techniques combined with large-scale multi-omics studies have enabled high-resolution multi-platform profiling of large cohorts of ACC and have provided novel insights into its molecular pathogenesis (15). Comprehensive multi-omics studies have greatly enhanced our understanding of the molecular mechanisms underlying adrenocortical carcinogenesis. Notably, the TCGA-ACC project has defined three distinct molecular subtypes, COC1, COC2, and COC3. COC1 has the best prognosis, while COC2 has an intermediate prognosis, and COC3 exhibits the worst prognosis. This molecular classification refines our understanding of ACC through multi-omics integration and further identifies subtype-specific therapeutic vulnerabilities (15). Genetic alterations identified so far in ACC span key pathways, among which are Wnt/β-catenin signaling (16, 17), insulin-like growth factor 2/insulin-like growth factor 1 receptor (IGF2/IGF1R) pathway (18–21), cell cycle and p53-mediated apoptosis (16, 22), telomere maintenance (23, 24), cAMP-PKA signaling (25, 26), covering multiple steps involved in transcription and translation. The ACC multi-omics studies have discovered unique molecular subtypes, potential therapeutic targets, and prognostic biomarkers with research covering mRNA, miRNA, DNA methylation, proteomics, and metabolomics (27–30). The influence of the immune microenvironment on drug sensitivity has also been investigated (31). Xenografts and genetically engineered mouse models are utilized to evaluate anticancer therapies, contributing to a more profound understanding of ACC pathogenesis (32, 33). This review will discuss recent findings in genomics, progress in targeted therapies, and ongoing research in immunotherapy, with the aim of specifying the therapeutic landscape and future directions in the personalized treatment, with particular attention to ACC in adults.

## 2 Genomic and molecular mechanisms

### 2.1 Core genetic pathways in ACC pathogenesis

Comprehensive genomic studies of ACC have revealed recurrent mutations in critical pathways, including Wnt/β-catenin (ZNRF3, CTNNB1), p53-driven apoptosis and cell cycle regulation (CDKN2A, CDK4, RB1, TP53), chromatin remodeling and telomere stabilization (MEN1, DAXX, TERT, TERF2, ATRX), cAMP-PKA signaling (PRKAR1A), and the IGF2/IGF1R axis, all of which contribute to dysregulated cell proliferation, impaired apoptosis, and an unfavorable prognosis (9).

The IGF2/IGF1R axis is crucial in the progression of ACC, with IGF2 overexpression occurring in nearly 90% of cases (30). This overexpression drives tumor cell proliferation by stimulating IGF1R and subsequent signaling pathways, including the PI3K/AKT/mTOR and RAS/RAF/MEK/ERK pathways, and is often associated with epigenetic changes at the 11p15 locus (30, 34). This dysregulation contributes to tumor growth and resistance to apoptosis, positioning IGF2/IGF1R as an important target for therapy in ACC. The Wnt/β-catenin pathway (8, 9, 15, 30) is implicated in up to 54% of ACC cases and is crucial in the development of ACC, with CTNNB1 mutations causing β-catenin accumulation that drives cell proliferation and survival. ZNRF3 mutations, which disrupt Wnt pathway inhibition, further enhance β-catenin signaling in ACC. TP53 mutations in ACC disrupt the p53 tumor suppressor pathway, leading to uncontrolled cell proliferation, DNA damage accumulation, and increased genomic instability; these mutations are notably common in Li-Fraumeni syndrome and sporadic ACC. Additionally, alterations in cell cycle regulators such as CDKN2A, RB1, CDK4, and CCNE1 drive unchecked proliferation, with RB1 or CDKN2A loss removing growth checkpoints and CDK4 and CCNE1 amplification accelerating the cell cycle, often correlating with more aggressive ACC and poorer prognosis (9, 15, 30, 35).

### 2.2 Growth factor and cytokine-mediated signaling pathways

Multiple growth factors and cytokines, including transforming growth factor-α (TGF-α) (35), transforming growth factor-beta 1 (TGF-β1) (35), vascular endothelial growth factor (VEGF) (35, 36), fibroblast growth factor (FGF-2) (9, 35), and several interleukins (35), play essential roles in ACC progression. These molecules bind to tyrosine kinase-coupled receptors (TKRs) on cell surfaces, activating signaling cascades that regulate critical cellular processes. Specifically, TGF-α and TGF-β1 contribute to cell proliferation and immune modulation, aiding tumor growth and evasion of immune responses. VEGF primarily supports angiogenesis, which supplies nutrients to the tumor and is crucial for sustained growth and potential metastasis. FGF-2, along with VEGF, enhances cell survival and proliferation, further promoting tumor aggressiveness. The combined effect of these pathways in ACC leads to increased cellular resistance to apoptosis, heightened angiogenic potential, and an enhanced capacity for metastatic spread, which collectively worsen the prognosis.

### 2.3 DNA damage repair mechanisms

Alterations in DNA damage repair (DDR) pathways are frequently detected in ACC, affecting various pathways crucial for maintaining genomic stability. Notable mutations have been identified in genes responsible for damage sensing (such as ATR, ATM, CHEK2), mismatch repair (including MLH1, MSH2, MSH6), and homologous recombination (such as BRCA1, BRCA2, RAD51) (8, 9). These mutations disrupt essential DDR pathways, leading to DNA damage accumulation, genomic instability, and promoting tumor progression. Additionally, germline mutations in mismatch repair (MMR) genes link certain ACC cases to Lynch syndrome, a hereditary cancer syndrome. Studies, including those by TCGA, have identified additional driver mutations in tumor suppressor genes like NF1 and MLL4, further compromising DDR functions.

### 2.4 Chromatin remodeling and epigenetic modifications

Chromatin remodeling and epigenetic changes-such as DNA methylation at CpG islands, histone modification, and telomere maintenance-play key roles in tumor progression and prognosis (9, 15). Abnormal DNA methylation, including high levels of CpG island methylator phenotype (CIMP), results in the inactivation of tumor suppressor genes and is associated with poor outcomes. TERT promoter mutations increase telomerase expression, bypassing cellular senescence, while mutations in genes like EZH2, ATRX, and DAXX alter chromatin structure and transcription, promoting tumor growth and progression (9). Blocking chromatin remodeling enzymes, including histone-lysine N-methyltransferases KMT2A–KMT2D, menin, as well as histone-lysine N-methyltransferase SETD2, may offer a promising approach for targeted therapy.

### 2.5 Non-coding RNA and post-transcriptional regulation

In ACC, non-coding RNAs, including microRNAs (such as miR-483-5p) and long non-coding RNAs (like H19 and UCA1), participate in the regulation of gene expression post-transcription, impacting tumor aggressiveness, proliferation, and survival (9, 37). Dysregulated microRNAs often target tumor suppressor genes or oncogenes, promoting cancer progression, while lncRNAs influence pathways that support tumor growth and resistance to apoptosis.

## 3 Targeted therapy for ACC

Mitotane, a DDT derivative with adrenal toxicity, inhibits Sterol O-Acyltransferase 1 (SOAT1) in ACC cells, disrupting cholesterol balance and enhancing oxidative stress sensitivity to suppress tumor growth. Additionally, it induces cellular necrosis, inhibits steroidogenesis by targeting CYP11A1 and CYP11B1, and strongly induces CYP3A4, potentially reducing the efficacy of concurrent medications (38). Clinical trials, including the ADIUVO trial (39–41), are investigating the role of mitotane in post-surgical patients. Although cytotoxic chemotherapy combined with mitotane has shown efficacy in advanced cases, its objective response rate (ORR) in advanced ACC is limited to around 24% (5, 42). Additionally, these treatment regimens often lead to significant toxicities, particularly affecting the gastrointestinal and neurological systems, with symptoms such as nausea, vomiting, and headache, which may restrict their long-term use. Given these limitations, the investigation of molecular targeted therapies offers a promising avenue for enhancing personalized treatment strategies for ACC.

### 3.1 Inhibitors of the IGF-1R and mTOR pathways

IGF1R inhibitor NVP-AEW541 showed inhibition of tumor cell growth in NCI-H295R and RL251 ACC models, with an enhanced effect when used in combination with mitotane (43). Everolimus (RAD-001), an mTOR inhibitor, has been shown to reduce tumor cell growth both *in vitro* and *in vivo* in xenograft models using NCI-H295R cells (44). Early clinical attempt of IGF1R inhibitors, including linsitinib (OSI-906) and cixutumumab (IMC-A12), have demonstrated some preliminary activity against ACC, including partial responses in certain cases (18, 45). A phase 1 study (46) demonstrated the combination therapy of cixutumumab and temsirolimus (an mTOR inhibitor) resulted in extended disease stability, with 42% patients maintaining stability for over 6 months; however, limitations such as limited overall efficacy, significant toxicities (e.g., grade 4 hyperglycemia and multiorgan failure), and potential patient selection bias have hindered the continued development of these therapies for advanced ACC.

### 3.2 Inhibitors targeting Wnt/β-catenin signaling

Preclinical studies targeting Wnt/β-catenin signaling, involving small-molecule inhibitor PKF115-584 to block the TCF/β-catenin complex and doxycycline-triggered β-catenin suppression via shRNA, demonstrated reduced tumor cell proliferation and triggered apoptosis in cell culture studies, along with full inhibition of tumor growth *in vivo*. Nonetheless, the clinical efficacy of Wnt pathway inhibitors has yet to be fully established, and additional research is required to evaluate their potential benefits and limitations in treating ACC (9).

### 3.3 Inhibitors of tyrosine kinase receptors

Research into tyrosine kinase inhibitors (TKIs) for ACC has shown varied outcomes across different agents. Cabozantinib, which inhibits multiple kinases including VEGF, AXL, c-MET, and RET, demonstrated promising efficacy in a phase 2 trial with an overall survival of 16 weeks in advanced ACC patients, suggesting it may be a viable option following failure of other treatments (47). In a phase 2 trial involving advanced ACC patients, cabozantinib achieved a progression-free survival (PFS) rate of 72.2% at 4 months, with a median PFS of 6 months. Adverse events of grade 3 or higher were reported in 61% of patients and were considered manageable (48). Studies on Sunitinib, which targets VEGFR and PDGFR, indicated limited efficacy, with a PFS of less than 3 months in patients with refractory ACC, partially due to drug interactions with mitotane, which decreases TKI blood levels (49). Sorafenib, another TKI with activity against VEGFR and RAF kinase, was also tested in ACC but failed to produce sustained tumor control, especially when combined with chemotherapy, leading to early trial termination (50, 51). Other agents, such as Dovitinib (an FGFR inhibitor) and Axitinib (a VEGFR inhibitor), showed minimal impact in terms of objective response rates, with Dovitinib achieving only stable disease in less than a quarter of patients for a duration exceeding 6 months (36). Additionally, targeting both EGFR and IGF1R in ACC through combined TKI therapy (Erlotinib and IGF1R inhibitor NVP-AEW541) has been explored preclinically, showing enhanced tumor inhibition compared to single-agent approaches, highlighting the potential for synergistic effects in dual-targeted strategies (52).

### 3.4 PPARγ agonists

Agonists of peroxisome proliferator-activated receptor gamma (PPARγ), including pioglitazone and rosiglitazone, have demonstrated significant anti-tumor effects in ACC through multiple mechanisms in preclinical studies. These agents inhibit tumor cell proliferation and invasiveness, promote apoptosis, and reduce angiogenesis by downregulating pro-survival markers like VEGF and Bcl-2, additionally, by suppressing critical signaling pathways such as PI3K/AKT and extracellular signal-regulated kinase 1 and 2 (ERK1 and ERK2) (9, 53–55). In xenograft models, rosiglitazone treatment resulted in a marked reduction in tumor volume, decreased microvessel density, and increased expression of C-X-C motif chemokine ligand 12 (CXCL12), a signaling protein associated with lower malignancy and better survival (56).

### 3.5 Inhibitors targeting cell cycle and DNA damage repair

Nutlin-3a (RG7112), an MDM2 inhibitor, has shown efficacy in reducing tumor growth, inducing apoptosis, and inhibiting cellular proliferation and hormone production in ACC models, especially those with CTNNB1 mutations (57). Preclinical studies indicate that targeting polo-like kinase 1 (PLK-1) with selective inhibitors like BI-2536, alone or combined with MDM2 inhibition, effectively reduces cell viability, restores p53 function, and induces apoptosis in adrenocortical carcinoma (ACC) cells, suggesting a promising therapeutic approach for ACC (58, 59). CDK4/6 inhibitors, such as palbociclib and ribociclib (9, 60), have shown efficacy *in vitro* by reducing cell viability and inducing senescence or apoptosis in ACC cell lines. Additionally, poly (ADP-Ribose) Polymerase (PARP) inhibitors, such as rucaparib, olaparib, talazoparib and niraparib, have been investigated due to their potential synthetic lethality in DDR-deficient tumors, although their efficacy in ACC specifically requires further investigation. In summary, these studies highlight CDK4/6 inhibitors and DDR-targeting agents as potential therapies. Further clinical trials are required to validate their efficacy and safety.

### 3.6 Epigenetic modifiers and non-coding RNA therapies

Recent studies on ACC have focused on various epigenetic therapies, including histone deacetylase (HDAC) inhibitors (vorinostat) and DNA methylation inhibitors (decitabine), both of which have shown potential antitumor activity in preclinical models. Vorinostat demonstrated enhanced efficacy when combined with standard chemotherapy, while decitabine exhibited tumor-suppressive effects by reactivating silenced genes and reducing ACC cell proliferation, cortisol secretion, and invasiveness (61–64). The study emphasizes several miRNAs, including miR-483-5p, miR-139-5p, miR-100, miR-34a, miR-184, and miR-181b. Furthermore, long non-coding RNAs such as PRINS, RAD50, and HAND2 are also highlighted for their involvement in tumor progression and association with poor prognosis, suggesting their potential as therapeutic candidates in ACC (9).

### 3.7 Selective estrogen receptor modulators

Research into estrogen and progesterone pathway inhibition in ACC shows promising preclinical findings (9). Hydroxytamoxifen is an active metabolite produced from tamoxifen, which has been shown to increase FASL (Fas ligand, a pro-apoptotic factor) expression, leading to reduce *in vitro* cellular growth and decreased tumor progression in xenograft models. G-1, which is a nonsteroidal G-protein coupled estrogen receptor (GPER) agonist, demonstrated proliferation inhibition in ACC cells through stimulation of the ERK1/2 signaling pathway in both laboratory and animal models. The inverse agonist XCT790, which targets estrogen-related receptor alpha (ERRα), was shown to impair mitochondrial activity, leading to a decrease in ACC cell proliferation and promoting cell death both *in vitro* and in animal models.

### 3.8 Steroidogenesis-targeted therapies

Recent research has identified steroidogenesis inhibitors as promising therapies for ACC, aiming to block key enzymes and pathways involved in hormone production. The acyl-CoA cholesterol acyltransferase 1 (ACAT1) inhibitor, ATR-101, has demonstrated efficacy in preclinical studies by inducing apoptosis in ACC cells through mechanisms such as mitochondrial hyperpolarization, oxygen species (ROS) generation, and activation of endoplasmic reticulum (ER) stress (65, 66). Other compounds, including AC-45594 (an alkyloxyphenol) and OOP (an isoquinolinone), suppress ACC cell proliferation by targeting the transcription factor steroidogenic factor 1 (SF-1), which plays a crucial role in steroid synthesis and is often overexpressed in ACC (67).

## 4 Immunotherapy approaches in ACC

Interest in immunotherapy research has grown because of the limited efficacy that traditional treatments for ACC have shown. Most recently, attention has fallen on immune checkpoint inhibitors (ICIs), IL-13Rα2-targeting, and other forms of precision therapy aimed at a more tailored approach in the treatment of ACC. Specific features in ACC and its immune evasion mechanisms do continue to complicate successful immunotherapeutic approaches in this disease.

Immune checkpoint inhibitors, especially those targeting cytotoxic T-lymphocyte associated protein 4 (CTLA-4) and programmed cell death protein 1 (PD-1), proved effective in treating various solid tumors, such as melanoma, renal cell carcinoma and non-small cell lung cancer. A number of ACC trials with PD-1 inhibitors have been completed or are in progress, and the majority of those so far utilized pembrolizumab and avelumab, but despite inducing tumor-suppressive effects in some patients, the overall efficacy was limited. Results from single-agent studies, for instance, were quite modest. A phase 2 study revealed that pembrolizumab led to a 23% response rate and a 52% rate of disease control in individuals with advanced adrenal cortex carcinoma, accompanied by a median survival duration of 24.9 months and tolerable safety in microsatellite-stable patients (68). Ipilimumab and nivolumab, when used together, proved effective in patients with refractory metastatic ACC, achieving a 19% immune response rate and 33% clinical benefit rate, with PFS up to 57 months (69). Thus, some patients with ACC do seem to benefit from ICIs, but these drugs do not improve the survival of most appreciably. A recent study revealed that the combination of camrelizumab and apatinib exhibited substantial antitumor effects in previously treated advanced ACC patients, achieving an objective response rate of approximately 50% and a median PFS duration of 12.6 months, with manageable toxicity (70). Exploratory analysis indicated changes in the immune microenvironment, including increased CD8+ and CXCR3+ T cells, reduced immunosuppressive CD4+ T cells, and higher clonal overlap between tumor-infiltrating and circulating T cells, suggesting that these immune alterations may be associated with a favorable treatment response. While ICIs show potential benefits for some ACC patients, further research is needed to address sample size, limited effectiveness, drug toxicity, patient selection in trials, and combination therapy strategies.

Thus, IL-13Rα2 has become an attractive target. Very highly overexpressed in the cells of ACC but minimally in normal adrenal tissue, IL-13Rα2 is an attractive target for immunotherapy. IL-13-PE, a chimeric protein comprising IL-13, specifically targets ACC cells that express IL-13Rα2. In the Phase 1 trial of IL-13-PE, three out of six IL-13Rα2-positive ACC patients developed disease stability lasting for 2 to 5.5 months, suggesting that IL-13Rα2-targeting immunotherapy may have antitumor efficacy and could be a novel treatment for ACC in the future (71). Research is also being conducted on chimeric antigen receptor T-cells (CAR-T) that target IL-13Rα2 (72). This method involves engineering T cells from a patient to recognize and attack ACC cells expressing IL-13Rα2. Preclinical results have demonstrated promising antitumor efficacy, indicating that IL-13Rα2-targeting therapies may be further developed to play a more important role in ACC immunotherapy.

## 5 Conclusion and future directions

**Figure 1:**
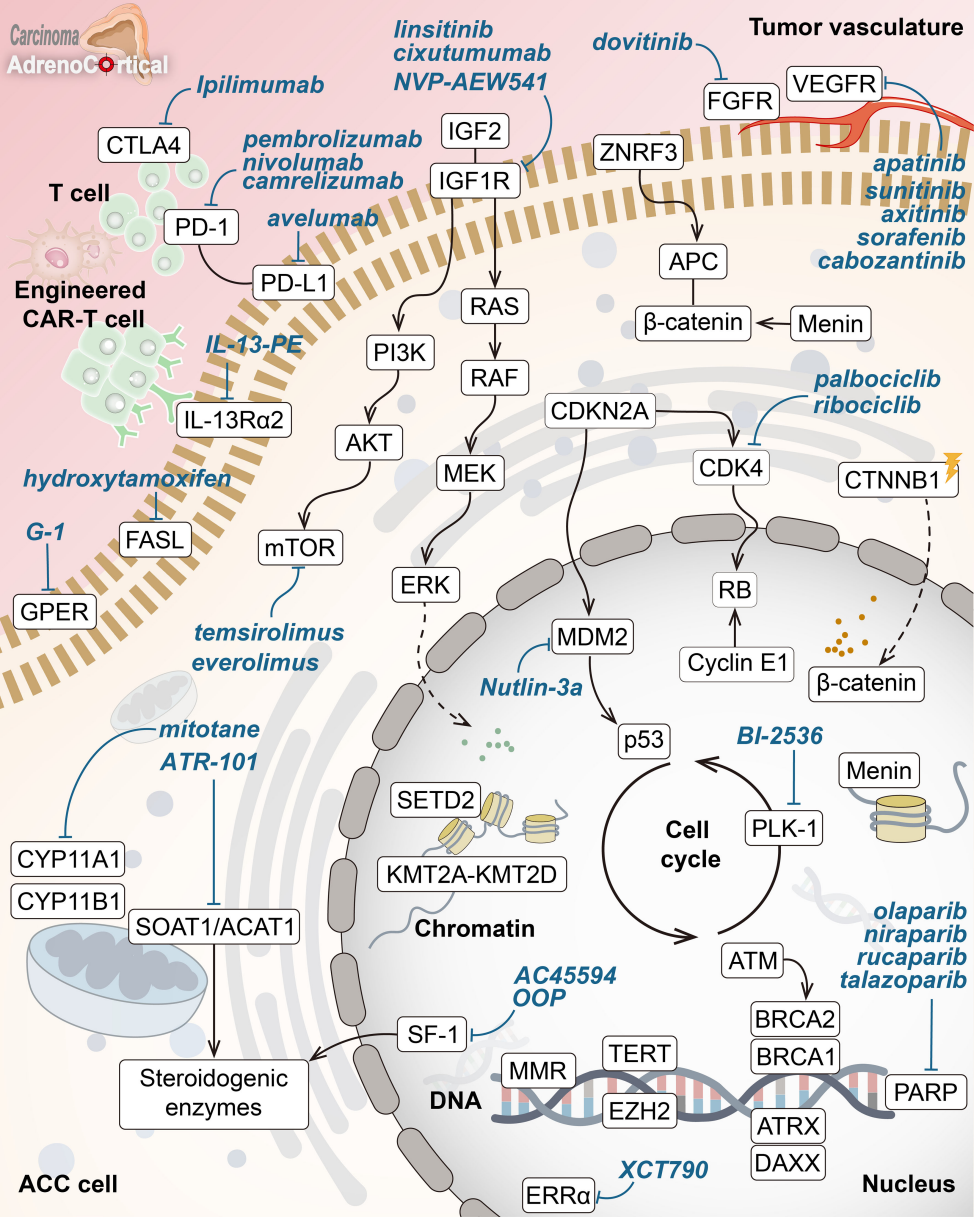
Key molecular drivers and drug targets in adrenocortical carcinoma. Overview of core pathways in adrenocortical carcinoma (ACC) pathogenesis with potential targeted therapies (in blue). Key hallmarks include abnormal Wnt/β-catenin signaling, disrupted cell cycle (p53-RB, CDK4), chromatin remodeling inhibition (menin, KMT2A-KMT2D, SETD2), DNA repair defects (MMR enzymes, ATM, BRCA1/2, PARP), altered telomere maintenance (ATRX, DAXX, TERT), and abnormal steroid metabolism. Highlighted treatments target IGF1R, mTOR, VEGFR, β-catenin, SOAT1, and ERRα, along with immune checkpoint inhibitors and other pathways.

Advances in molecular and genetic research have considerably enlightened the rare and aggressive nature of adrenocortical carcinoma over recent years. Molecular-targeted therapies have also promised a new ray of hope through specific inhibitions crucial for the growth and differentiation of ACC cells. A overview of key molecular drivers and drug targets in ACC is shown in **Figure 1**. However, the low efficacy detected in clinical trials so far underlines that big challenges are yet to be overcome in the identification of reliable molecular drivers and effective therapeutic outcomes. Current limitations include bypass pathway activations, lack of precise molecular targets for patient stratification, and insufficient response to single-agent therapies. These findings emphasize that more detailed knowledge about the molecular mechanism of ACC is necessary to guide individualized treatment options.

Future treatment strategies for ACC are anticipated to combine molecular diagnostics, targeted therapies, and immunotherapy. The constant identification of actionable biomarkers and genetic signatures provides hope for diagnostics, as these facilitate the prompt identification and classification of patients who may respond to targeted treatments. Biomarker-driven clinical trials require emphasis in ongoing research to bring personalized approaches to ACC management. Lastly, although immunotherapy has challenges like low mutational burden and immune evasion mechanisms, the approach seems promising when combined with other therapies. Preliminary data indicate that ICIs may provide clinical benefit when combined with VEGF inhibitors or other targeted therapies. It is also multidisciplinary and a team approach. Centers of excellence and international collaboration in research can accelerate the transform of basic research to clinical applications. The use of multi-omics technologies and next-generation sequencing may, in the future, provide a better insight into ACC-specific biological features and enable the drug repositioning active in other cancers for ACC treatment. In addition, some novel therapeutic approaches or concepts, such as some artificial intelligence-guided, voluntary exercise-combined or nanomaterials-based therapies, have provided potential directions for ACC treatment in future, yet rigorous preclinical validation and clinical trials are still warranted to confirm their clinical perspective and therapeutic efficacy (73–77). Further research should be targeted toward resistance mechanisms elucidation, optimization of combination regimens, and early detection and therapeutic strategies that could potentially enhance survival rates and overall well-being for patients with ACC.

## Glossary

ACC: adrenocortical carcinoma
ATR: ataxia telangiectasia and Rad3 related
ATM: ataxia telangiectasia mutated
BRCA1: breast cancer 1
BRCA2: breast cancer 2
CHEK2: checkpoint kinase 2
CTLA-4: cytotoxic T-lymphocyte associated protein 4
CYP11A1: cytochrome P450 family 11 subfamily A member 1
CYP11B1: cytochrome P450 family 11 subfamily B member 1
CYP3A4: cytochrome P450 family 3 subfamily A member 4
DAXX: death domain associated protein
DDR: DNA damage repair
EDP-M: etoposide, doxorubicin, and cisplatin with mitotane
EZH2: enhancer of zeste homolog 2
FDA: Food and Drug Administration
FGF-2: fibroblast growth factor 2
ICIs: immune checkpoint inhibitors
IGF1R: insulin-like growth factor 1 receptor
IGF2: insulin-like growth factor 2
IL-13Rα2: interleukin 13 receptor alpha 2
KMT2A–KMT2D: Lysine (K) Methyltransferase 2A–2D
lncRNA: long non-coding RNA
miRNA: microRNA
MLH1: MutL homolog 1
MLL4: mixed lineage leukemia 4
MSH2: MutS homolog 2
MSH6: MutS homolog 6
mTOR: mechanistic target of rapamycin
NF1: neurofibromin 1
PD-1: programmed cell death protein 1
PPARγ: peroxisome proliferator-activated receptor gamma
RAD51: RAD51 recombinase
SETD2: SET Domain Containing 2
TCGA: The Cancer Genome Atlas
TERT: telomerase reverse transcriptase
TGF-α: transforming growth factor-alpha
TGF-β1: transforming growth factor-beta 1
TKI: tyrosine kinase inhibitor
VEGF: vascular endothelial growth factor
